# Analysis of lineage-specific *Alu* subfamilies in the genome of the olive baboon, *Papio anubis*

**DOI:** 10.1186/s13100-018-0115-6

**Published:** 2018-03-19

**Authors:** Cody J. Steely, Jasmine N. Baker, Jerilyn A. Walker, Charles D. Loupe, The Baboon Genome Analysis Consortium, Mark A. Batzer

**Affiliations:** Department of Biological Sciences, Louisiana State University, 202 Life Sciences Bldg., Baton Rouge, LA 70803 USA

**Keywords:** *Alu*, Subfamily, Papio, Baboon

## Abstract

**Background:**

*Alu* elements are primate-specific retroposons that mobilize using the enzymatic machinery of L1 s. The recently completed baboon genome project found that the mobilization rate of *Alu* elements is higher than in the genome of any other primate studied thus far. However, the *Alu* subfamily structure present in and specific to baboons had not been examined yet.

**Results:**

Here we report 129 *Alu* subfamilies that are propagating in the genome of the olive baboon, with 127 of these subfamilies being new and specific to the baboon lineage. We analyzed 233 *Alu* insertions in the genome of the olive baboon using locus specific polymerase chain reaction assays, covering 113 of the 129 subfamilies. The allele frequency data from these insertions show that none of the nine groups of subfamilies are nearing fixation in the lineage.

**Conclusions:**

Many subfamilies of *Alu* elements are actively mobilizing throughout the baboon lineage, with most being specific to the baboon lineage.

**Electronic supplementary material:**

The online version of this article (10.1186/s13100-018-0115-6) contains supplementary material, which is available to authorized users.

## Background

*Alu* elements are non-autonomous, non-long terminal repeat (non-LTR) retroposons found in high copy numbers in the genomes of primates [1, 2]. They consist of a left and right monomer separated by an A-rich middle linker region, along with an A-rich tail at the 3′ end of the element [3, 4]. These elements mobilize using proteins encoded by LINE-1 elements (L1 s), via a retrotransposition mechanism termed Target Primed Reverse Transcription (TPRT) [5, 6]. This mechanism allows for the creation of new copies of the element and for these copies to be inserted at novel locations in the genome (Reviewed in [1, 2, 7, 8]). *Alu* elements are short (approximately 300 base pairs (bp)), making them relatively easy to amplify and genotype via polymerase chain reaction (PCR) and agarose gel electrophoresis. They have also been useful for phylogenetic and population genetics analyses, as they are nearly homoplasy free and the ancestral state of an element is known to be the absence of that insertion [9]. Hence, they have been used in a number of molecular studies over the last few decades [10–25].

*Alu* elements can be broken down into subfamilies based on diagnostic mutations [26–29]. There are 3 major subfamilies of *Alu* elements: J, S, and Y [30]. These major subfamilies of *Alu* elements can be further expanded based on diagnostic mutations that they have accrued over millions of years [31]. Some subfamilies of elements can be shared within a number of closely related taxa, but other recent studies have identified elements that are unique to only a particular species or genus [15, 32]. This parallel evolution of *Alu* subfamilies results in each primate lineage having its own network of recently integrated *Alu* subfamilies [2]. The recent work of the Baboon Genome Analysis Consortium has revealed a great deal of information about the content of the baboon genome, including a much higher rate of *Alu*Y mobilization than seen in other primates (Rogers et al: The comparative genomics, epigenomics and complex population history of Papio baboons. In Preparation). Previous work on *Alu* elements in baboons has already been informative for population structure, species identification, and as a polymorphic marker for hybrid individuals in the field [33–35].

Baboons (genus *Papio*) are found throughout sub-Saharan Africa in distinct ranges with slight overlap. There are six species of baboons that are part of most recent studies, including: yellow baboon (*Papio cynocephalus),* olive baboon (*Papio anubis),* hamadryas baboon (*Papio hamadryas),* guinea baboon (*Papio papio),* chacma baboon (*Papio ursinus),* and the kinda baboon (*Papio kindae).* These six baboons are largely differentiated based on morphological differences (size, pelage coloration), as well as geographic range, dispersal, and social traits (Rogers et al: The comparative genomics, epigenomics and complex population history of Papio baboons. In Preparation) [36–38]. Though they differ in the above ways, many of these species are also known to be interfertile, with a number of studies examining their active hybrid zones [39–43]. Given their anatomical and physiological similarity to humans, baboons have been used for a number of medical studies, and have proven particularly valuable for cardiovascular studies [44–46]. In this study, due to the rapid mobilization of *Alu*Y elements in baboons reported in Rogers et al., and the recent utility of *Alu* elements for studies in baboons, we aimed to analyze the expansion of *Alu* subfamilies in the genome of the olive baboon, *Papio anubis*.

## Methods

### Ascertainment of baboon-specific *Alu* elements

Loci were ascertained by first using RepeatMasker [47] on the reference genome of the olive baboon, *Papio anubis* (Panu_2.0). *Alu* elements were parsed out of the resulting RepeatMasker file. The sequence of each full length (starts at or before position 4 in the element and ends after position 266) *Alu*Y insertion, along with 500 bases of flanking in 5′ and 3′ direction of the *Alu* element, was compared to the rhesus macaque (rheMac8) and human (hg19) reference genomes using BLAT [48]. We then compared the resulting BLAT files for any locus that had an appropriate gap size in the genomes that would indicate an insertion that was only present in the genome of the olive baboon.

### COSEG analysis & network figure creation

Our *Papio* specific set of *Alu* elements was aligned to the *Alu*Y consensus sequence [49] using cross_match (http://www.phrap.org/phredphrapconsed.html; last accessed December 2017). The data set was then analyzed via COSEG (www.repeatmasker.org/COSEGDownload.html; last accessed November 2017) to determine subfamilies. The middle A-rich region of the *Alu*Y consensus sequence was omitted while tri and di segregating mutations were considered. Using these criteria, a set of ten or more identical sequences was considered an individual *Alu* subfamily. A network analysis of all subfamilies of *Alu* elements identified by COSEG was created by uploading the source and target subfamily information into Gephi (v0.9.1) [50].

### Oligonucleotide primer design

Primers were designed using an in house Python script that utilized BLAT, MUSCLE (v3.8.31) [51], and a modified version of Primer3 [52]. Briefly, target sequences acquired from the genome of the reference olive baboon and orthologous sequences were found in human (hg19), chimpanzee (panTro4), and rhesus macaque (rheMac8) using BLAT. These sequences were then aligned using MUSCLE, and potential oligonucleotide primer locations were identified using Primer3. Oligonucleotide primers for PCR were ordered from Sigma Aldrich (Woodlands, TX). A complete list of PCR primers and genomic locations is available in Additional file 1 (worksheet “PCR Primer Information”).

### Polymerase chain reaction assays

The PCR format and the DNA samples used for PCR assays are reported in Additional file 1 (worksheet “DNA panel”). We attempted to analyze at least 5 *Alu* insertions from each of the 9 main groups of *Alu* subfamilies in this report. PCR amplification was performed in 25 μL reactions that contained 25–50 ng of template DNA, 200 nM of each primer, 1.5 mM MgCl_2_, 10× PCR buffer, 0.2 mM deoxyribonucleotide triphosphates and 1 unit of *Taq* DNA polymerase. The PCR protocol is as follows: 95 °C for 1 min, 32 cycles of denaturation at 94 °C for 30 s, 30 s at a 57 °C annealing temperature, and extension at 72 °C for 30 s, followed by a final extension step at 72 °C for 2 min. Gel electrophoresis was performed on a 2% agarose gel containing 0.2 μg/mL ethidium bromide for 60 min at 200 V. UV fluorescence was used to visualize the DNA fragments using a BioRad ChemiDoc XRS imaging system (Hercules, CA). Loci that did not amplify clearly were re-run using the JumpStart *Taq* DNA Polymerase kit from Sigma Aldrich.

### Nucleotide model selection & tree design

The consensus sequence of each identified *Alu* subfamily was input into jModelTest-2.17 [53] for analysis and to determine the best model of nucleotide evolution for the data set. The Akaike Information Criterion (AIC) model selected was Trn + G, which includes variable base frequencies with equal transversion rates, but variable transition rates. The Bayesian Information Criterion (BIC) selected was TrNef+G, which includes equal base frequencies, equal transversion rates, but variable transition rates.

The AIC model selected by jModelTest was input into PhyML [54], which was used to create the maximum likelihood tree, and the BIC model selected by jModelTest was input into BEAST (v2.4.6) [55], which was used to create the Bayesian tree. The TreeAnnotator program in BEAST was then used to summarize the information from the BEAST output, and FigTree (v1.4.3) (http://tree.bio.ed.ac.uk/software/figtree/) was then used to visualize and create figures for both the maximum likelihood and Bayesian trees.

## Results

### COSEG analysis and alignment

**Fig. 1:**
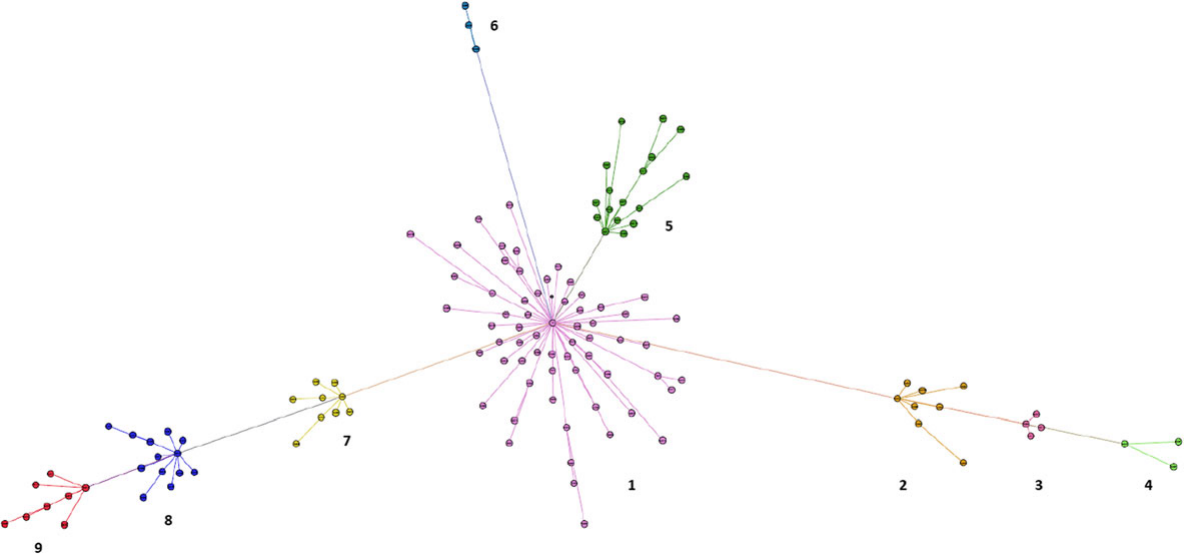
Network analysis of the COSEG assigned subfamilies, with each identified subfamily as a single node. Related subfamilies are clustered together, are connected by lines, and all branch out from the central node (labeled Cluster 1, shown in purple). Line length between subfamilies is not indicative of number of mutations or evolutionary time between subfamilies

In this study, through the use of python scripts and BLAT comparisons to the genomes of human and rhesus macaque, we ascertained and examined a total of 28,114 baboon-specific, full-length *Alu* element insertions. We used the genome of the rhesus macaque as an outgroup for comparison, as the *Papio* lineage diverged from the macaque lineage roughly 8 million years ago and our primary interest was finding elements that were unique to the genome of the baboon. Cross_match (see methods) was used for pairwise alignment, and these insertions were uploaded and analyzed by COSEG, producing 129 distinct *Alu* subfamilies for further investigation. The number of elements matching each consensus sequence produced by COSEG can be found in Additional file 1 (Worksheet “Subfamily Counts”). The consensus sequence for each of these 129 *Alu* subfamilies is available in Additional file 2. These subfamilies were uploaded into Gephi for visualization (Fig. 1 with a high resolution PDF available in Additional file 3). There are 9 major clusters of *Alu* elements (assigned a cluster number 1–9) that radiate from a single, central node (Subfamily 0) as shown in Fig. 1. Our subfamilies expand in a star-burst pattern, similar to bush-like shaped expansions of *Alu* elements previously reported [56]. It is important to note that the subfamily names assigned by the COSEG output are random, and not numerically ordered to be indicative of the network of source and offspring elements.

**Fig. 2:**
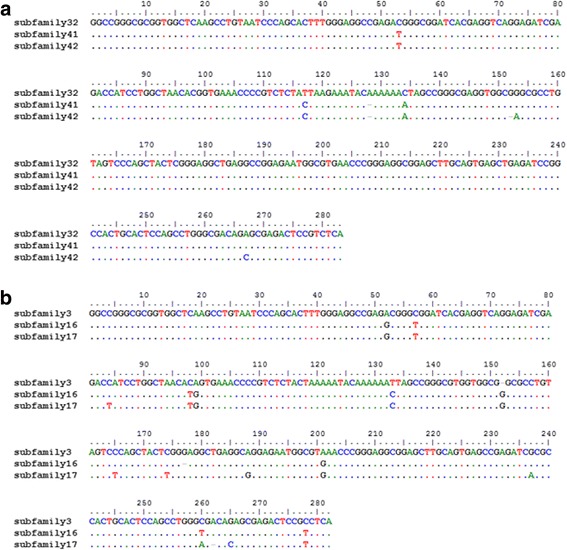
Alignment of consensus sequence for *Alu* subfamilies positioned in the central node of radiating clusters illustrated in Fig. 1. **a**. Alignment of central subfamilies from Clusters 2, 3, and 4. This alignment shows the accumulation of diagnostic mutations that have occurred over time. Subfamily 41 (Cluster 3) acquired new mutations when compared to Subfamily 32 (Cluster 2), and Subfamily 42 (Cluster 4) shares diagnostic mutations with Subfamily 41 while acquiring additional mutations. **b**. Alignment of central subfamilies from Clusters 7, 8, and 9 showing a similar acquisition of diagnostic mutations over time. Subfamily 16 (Cluster 8) acquired mutations when compared to Subfamily 3 (Cluster 7), and Subfamily 17 (Cluster 9) continued to acquire mutations when compared to Subfamily 16

To determine if the subfamilies were novel, we aligned the consensus sequences of the subfamilies that were produced by COSEG with one another and to *Alu* elements from RepBase [49] using MUSCLE (Fig. 2, and complete alignment in Additional file 2). The resulting MUSCLE output was visualized in BioEdit [57] to determine if these subfamilies were novel or had been previously discovered. We found that 127 of 129 subfamilies were newly discovered in this study, with only two of these subfamilies that had been previously identified. Subfamily 70 aligned to the consensus sequence of *Alu*Y, and Subfamily 0 aligned with the consensus sequence of *Alu*MacYa3, previously discovered in the genome of the rhesus macaque and reported in Repbase (Smit, A. F. AluMacYa3-SINE1 SINE from Macaca. Direct submission to Repbase Update (06-Sep-2005)). The central subfamily for Clusters 2, 3, and 4 (as numbered in Fig. 1) were aligned, along with the central subfamily for Cluster 7, 8, and 9 (Fig. 2). As the clusters radiate outward from Cluster 1, they accrue more mutations, allowing for the visualization of subfamily specific evolution.

**Fig. 3:**
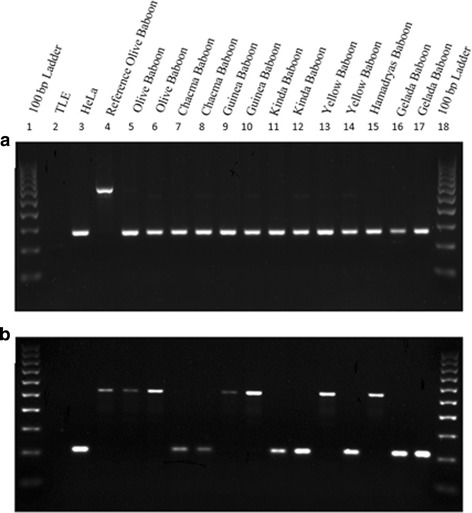
**a**. Agarose gel chromatograph of a polymorphic, olive baboon-specific *Alu* insertion (found at chr3:168089568–168,090,568; primer information can be found in Additional file 1 (Worksheet “PCR Primer Information”)). Each lane of the gel is labeled at the top of the image. The filled (insertion present) (~ 590 bp) site is seen only in the reference olive baboon individual (lane 4), and empty (insertion absent) sites (~ 275 bp) are seen in all other individuals. **b**. Agarose gel chromatograph of a polymorphic, *Papio*-specific *Alu* insertion (found at chr4:144885392–144,886,392; primer information can be found in Additional file 1 (Worksheet “PCR Primer Information”)). The empty site is found in lane 3 (HeLa, human control), lanes 7 and 8 (chacma baboons), lane 11 and 12 (kinda baboons), lane 14 (one yellow baboon), and lanes 16 and 17 (gelada baboons). The filled site can be seen in lanes 4–6 (olive baboons), 9 and 10 (guinea baboons), lane 13 (one yellow baboon), and lane 15 (hamadryas baboon). A list of DNA samples is available in Additional file 1, worksheet “DNA panel”

In order to confirm our computational findings, we designed oligonucleotide primers using an in-house Python script and analyzed 233 young (< 2% diverged from the consensus sequence) insertions through locus specific PCR and gel electrophoresis (Fig. 3). With these 233 assays, we were able to confirm the presence of 113 of our 127 (89%) novel subfamilies. We also successfully amplified at least five insertions from each of the nine major clusters shown in Fig. 1. The 14 subfamilies that were not successfully PCR validated were reviewed and we found that these loci were in repeat-rich genomic regions, limiting the effectiveness of these particular assays. Detailed information for each locus examined, as well as primer information and allele frequency data can be found in Additional file 1 (worksheets “PCR Primer Information” and “Genotypes”).

Full length *Alu* elements from each of the 9 clusters were identified and examined for divergence from the consensus sequence. We found a total of 19,888 full length elements that were classified by RepeatMasker to be members of novel subfamilies discovered in this study. 12,800 (~ 64%) of these *Alu* repeats were determined to be less than 2% diverged from their respective consensus sequence (Table 1). Elements that are less than 2% diverged from their consensus sequence are considered to be relatively young, as they have not accrued many mutations since their insertion [58, 59]. The percentage of elements less than 2% diverged from their consensus sequence varies from cluster to cluster, and though the sample size of elements that were analyzed by PCR was modest for some of the clusters, the allele frequency among the individuals of genus *Papio* for each cluster was far from fixation, ranging from ~ 40% to ~ 66% (Table 1).

**Table 1:** Number of elements from each of the nine clusters of *Alu* subfamilies

Cluster	Full Length Elements	< 2% Divergence from Consensus	Percent with less than 2% divergence	Number of successfully amplified loci	Allele Frequency
1	8076	4962	61.44	121	0.529995
2	1311	1046	79.79	14	0.553571
3	638	470	73.67	8	0.660173
4	477	436	91.40	6	0.594276
5	1494	1177	78.78	31	0.481322
6	2456	682	27.77	5	0.631944
7	862	452	52.44	10	0.418019
8	3262	2566	78.66	27	0.615822
9	1312	1009	76.91	11	0.414457

**Fig. 4:**
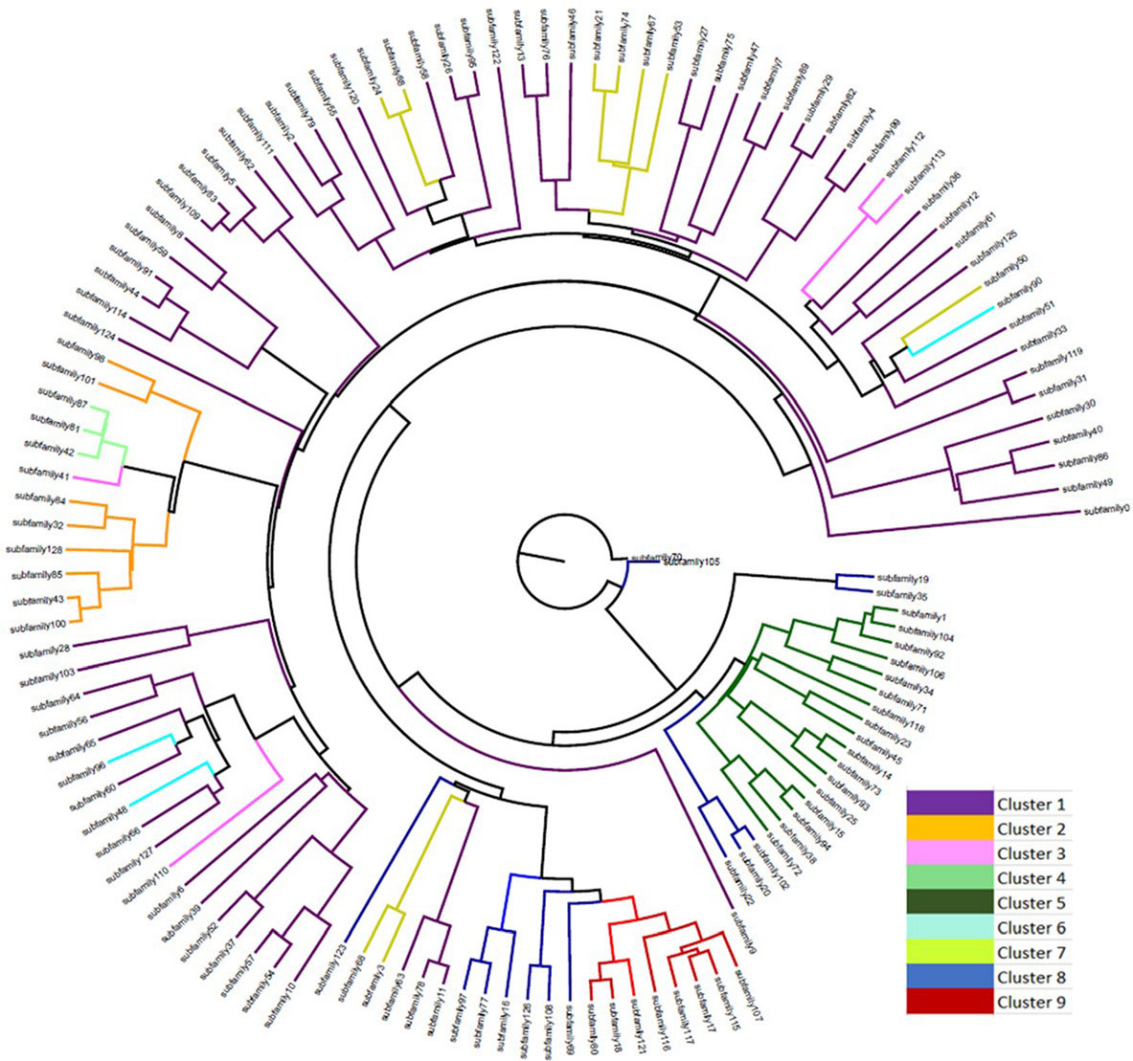
Bayesian tree created in BEAST, showing the relationship between subfamilies. The tree was rooted using the *Alu*Y consensus sequence (Subfamily 70). Each branch is colored based on the color of the cluster shown in Fig. 1 that the subfamily belongs to

jModelTest-2.17 was used to determine the best nucleotide model for creating a phylogenetic tree. Following the best model selected by jModelTest, we created both a Bayesian tree, using the BIC model chosen by jModelTest (Fig. 4 with a high resolution PDF available in Additional file 4), and a maximum likelihood tree using the AIC model chosen by jModelTest (Additional file 5). Both trees were rooted using subfamily 70, which was found to match the consensus sequence of *Alu*Y. The maximum likelihood tree shows many unresolved relationships between subfamilies; however, the general grouping of subfamilies is similar to those observed in Fig. 1. The Bayesian tree is more resolved and displays a defined branching pattern between all subfamilies. The Bayesian tree also displays subfamily relationships that are highly similar to the relationships determined by COSEG displayed in Fig. 1. Based on the Bayesian tree and alignments, we were able to determine the relative radiation of our *Alu* subfamilies and their possible derivatives.

## Discussion

The results of this study show an ongoing expansion of *Alu* elements in the *Papio* lineage, with more novel recently integrated subfamilies of *Alu* elements present than in any other previously analyzed human or non-human primate genome [15, 32, 60–68]. For comparison, only 14 lineage-specific *Alu* subfamilies were found in the genome of the rhesus macaque [61], and 46 *Saimiri-*specific *Alu* subfamilies were found in a recent analysis [32]. The 127 novel *Alu* subfamilies present in the baboon lineage supports the results of the Baboon Genome Analysis Consortium, which showed that the baboon lineage has undergone a rapid expansion of *Alu* elements (Rogers et al: The comparative genomics, epigenomics and complex population history of Papio baboons. In Preparation). Recent work also supports that the expansion of *Alu* elements is not unique to the olive baboon, but rather the *Papio* lineage as a whole [33, 35].

Of the nine clusters and 127 novel *Alu* subfamilies reported here, all of the elements (Fig. 1) appear to be derived from subfamilies discovered in the genome of Rhesus macaque [61]. The central subfamily of Cluster 1, which was determined to match the consensus sequence of *Alu*MacYa3, seems to be the source or parent to a large number of closely related subfamilies. All of the members of Cluster 6 are also very closely related to *Alu*MacYa3, showing only a small number of insertions or deletions near and moving into the middle A-rich region of the element. The central nodes of Cluster 2, 3, and 4, (subfamily 32, 41, and 42, respectively), as well as the surrounding, related subfamilies, all appear to be derived from *Alu* YRa1 [61]. The central nodes of Clusters 7, 8, and 9 (subfamily 3, 16, and 17, respectively) show a similar pattern, as they are likely derived from *Alu*YRa4 [61]. The apparent origin of these elements is not surprising given that the *Papio* lineage diverged from the *macaca* lineage roughly 8 million years ago (mya) (Rogers et al: The comparative genomics, epigenomics and complex population history of Papio baboons. In Preparation) Interestingly, the subfamilies present in Cluster 5 are similar to the *Alu*Yc (originally named Yd) [69] element present in humans, and the *Alu*YRb [61] family from the genome of Rhesus macaque. Each subfamily present in Cluster 5 (as well as the previously known similar elements in human and rhesus) shares the same 12 bp deletion in the left monomer of the element, supporting the prolonged activity and evolution of these closely related *Alu* subfamilies through multiple lineages [69].

All of the elements in our study expand out from a central subfamily, subfamily 0, originally found in the genome of the Rhesus macaque. The novel elements in our study follow the star-like or bush-like pattern of evolution (Fig. 1) as seen in a number of previous studies of *Alu* subfamily structure [56]. The expansion seen in these nine clusters supports the intermediate master gene model or stealth model, with multiple active elements leading to the expansion of new subfamilies (see Clusters 2–4, and Clusters 7–9) [56, 70]. The elements uncovered by this study appear to be quite young, with the majority of the full-length representatives of our novel subfamilies being under 2% diverged from their respective consensus sequences (see Table 1). The allele frequencies for polymorphic elements in each cluster also reflects this, as none of the clusters of closely related subfamilies appear to have reached fixation for the presence of the *Alu* element (Table 1) based on our small panel of *Papio* individuals (Additional file 1). The rapid radiation/expansion of genus *Papio*, which occurred only ~ 2.5 mya, likely contributes to this lack of allele fixation, along with gene flow from troop migration and hybridization occurring along active hybrid zones (Rogers et al: The comparative genomics, epigenomics and complex population history of Papio baboons. In Preparation) [33].

The Bayesian phylogenetic analysis (Fig. 4) largely supports the relationships displayed in the network analysis of COSEG results. However, it is important to note that the relationships shown in the network analysis reflect what COSEG has determined to be source and offspring elements, not phylogenetic relationships. Discrepancies between the phylogenetic tree and the network analysis are likely the result of two elements showing closer sequence identity, even if they did not come from the same “parent” node of the network analysis.

The recent findings of the Baboon Genome Analysis Consortium, along with other recent studies of mobile elements in the baboon genome have provided a great deal of new information (Rogers et al: The comparative genomics, epigenomics and complex population history of Papio baboons. In Preparation) [33, 35]. This study found 127 novel *Alu* element subfamilies, supporting the high *Alu* mobilization rate reported by the Baboon Genome Analysis Consortium (Rogers et al: The comparative genomics, epigenomics and complex population history of Papio baboons. In Preparation). It is important to note, however, that these elements are considered to be lineage-specific based on the genomic information currently available. Additionally, it is unlikely that the *Alu* elements uncovered in this study represent the loss of a particular element from the human or rhesus macaque genome as the precise deletion of an element is an exceedingly rare event [9]. As the number of sequenced primate genomes grows, and as sequencing quality continues to improve, it’s likely that new subfamilies may be discovered or that some of these newly reported subfamilies may be found in other closely related species. Future studies should attempt to determine underlying causes of rapid mobilization of transposable elements within the lineage. This increased duplication rate may extend to mobilization competent “master” elements including L1, or it may be caused by decreased activity of host defenses that have been shown to slow activity in humans [71–73].

## Conclusions

Overall, we identified 129 *Alu* subfamilies that were active in *Papio* baboons, with 127 of these insertions being baboon specific. This work reinforces that there has been extensive expansion of *Alu* elements and subfamilies within genus *Papio.*

## Additional files

Additional file 1: An excel file containing different worksheets for DNA samples and PCR format, PCR primers and genomic locations of amplified insertions, subfamily counts, and genotype data with allele frequency for each locus. (XLSX 78 kb)

Additional file 2: An alignment file of *Alu* subfamily consensus sequences. (FAS 114 kb)

Additional file 3: Higher resolution PDF of Fig. 1. (PDF 39 kb)

Additional file 4: Higher resolution PDF of Fig. 4. (PDF 16 kb)

Additional file 5: Maximum likelihood tree using the AIC model determined by jModelTest-2.17. (PNG 238 kb)

Additional file 6: List of the members of the Baboon Genome Analysis Consortium. (DOC 13 kb)

## Abbreviations

AIC: Akaike information criterion
BIC: Bayesian information criterion
Bp: Base pairs
L1: Long interspersed element-1
MYA: Million years ago
TPRT: Target primed reverse transcription
PCR: Polymerase chain reaction
