# Supplementary figures and images for: Analysis of lineage-specific Alu subfamilies in the genome of the olive baboon, Papio anubis
Source: Mob DNA. 2018 Mar 19;9:10. doi: 10.1186/s13100-018-0115-6 (PMC5858127; doi:10.1186/s13100-018-0115-6)

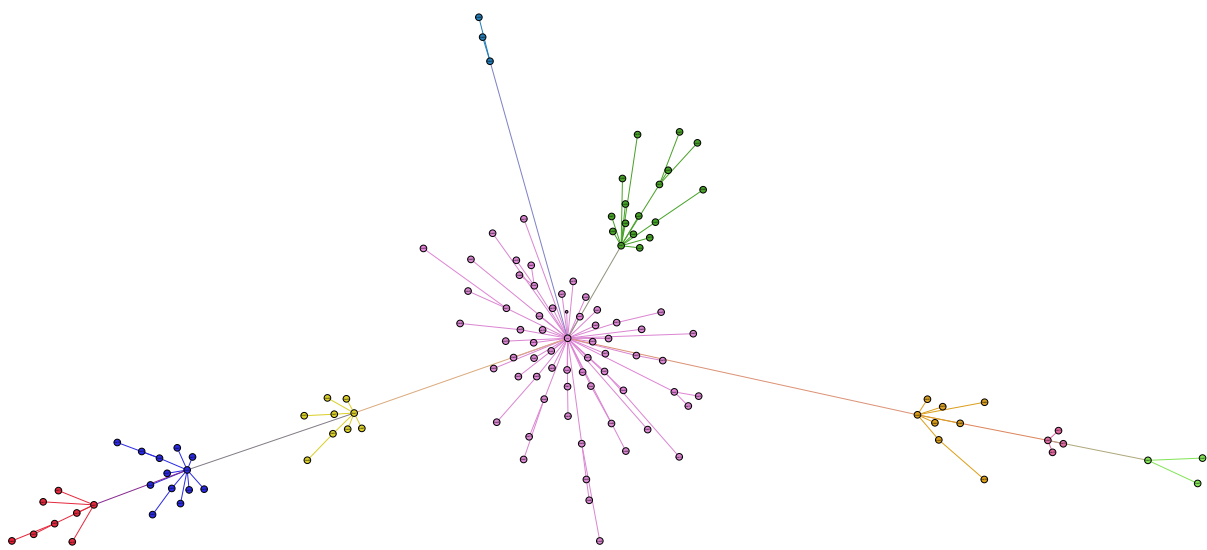

Supplement: Supplementary file 3 — Higher resolution PDF of Fig. 1. (PDF 39 kb) [file 13100_2018_115_MOESM3_ESM.pdf]

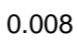

Supplement: Supplementary file 4 — Higher resolution PDF of Fig. 4. (PDF 16 kb) [file 13100_2018_115_MOESM4_ESM.pdf]

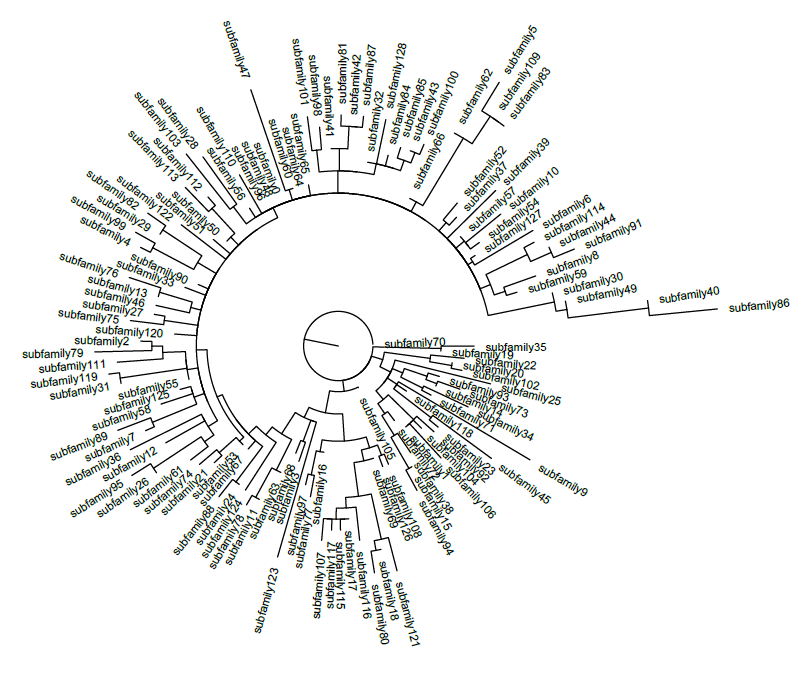

Supplement: Supplementary file 5 — Maximum likelihood tree using the AIC model determined by jModelTest-2.17. (PNG 238 kb) [file 13100_2018_115_MOESM5_ESM.png]
