# Supplementary material for: Analysis of lineage-specific Alu subfamilies in the genome of the olive baboon, Papio anubis
Source: Mob DNA. 2018 Mar 19;9:10. doi: 10.1186/s13100-018-0115-6 (PMC5858127; doi:10.1186/s13100-018-0115-6)
Supplement: Supplementary file 6 — List of the members of the Baboon Genome Analysis Consortium. (DOC 13 kb) [file 13100_2018_115_MOESM6_ESM.doc]

**Author List: The Baboon Genome Analysis Consortium**

Jeffrey Rogers^1,2^, R. Alan Harris^1,2^, Muthuswamy Raveendran^1^, Yue Liu^1^, Shwetha Murali^1*^,

Tauras P. Vilgalys^3^, Jerilyn A. Walker^4^, Miriam K. Konkel^4^, Vallmer E. Jordan^4^, Cody J. Steely^4^, Thomas O. Beckstrom^4^, Gregg W.C. Thomas^5^, Kymberleigh A. Pagel^6^, Vikas Pejaver^6^, Claudia R. Catacchio^7^, Nicoletta Archidiacono^7^, Mario Ventura^7^, Alessia Marra-Campanale^7^, Antonio Palazzo^7^, Oronzo Capozzi^7^, Archana Raja^8^, John Huddleston^8^, Veronica Searles Quick^9^, Anis Karimpour-Fard^9^, Dominik Schrempf^10^, Marc de Manuel Montero^11^, Konstantinos Billis^12^, Fergal J. Martin^12^, Matthieu Muffato^12^, Georgios Athanasiadis^13^, Christina Bergey^14^, Andrew Burrell^15^, Jade Cheng^13^, Laura Cox^16^, James Else^17^, Yi Han^1^, Gisela H. Kopp^18,19^, Maximilian Kothe^20^, Kalle Leppälä^13^, Angela Noll^19^, Jera Pecotte^21^, Lenore Pipes^22^, Karen Rice^21^, Christopher E. Mason^22^, Todd Disotell^15^, Jane Phillips-Conroy^23^, Lutz Walter^20^, Kasper Munch^13^, Thomas Mailund^13^, Mikkel Schierup^13^, Carolin Kosiol^10^, Tomas Vinar^24^, James M. Sikela^9^, Dietmar Zinner^18^, Christian Roos^19^, Clifford J. Jolly^15^, Predrag Radivojac^6^, Roscoe Stanyon^25^, Mariano Rocchi^7^, Evan E. Eichler^8,26^, Bronwen Aken^12^, Matthew W. Hahn^5^, Mark A. Batzer^4^, Tomas Marques-Bonet^11^, Jenny Tung^3^, Donna M. Muzny^1^, Richard A. Gibbs^1,2^, Kim C. Worley^1,2^

**Author Affiliations by Institution**

^1^Human Genome Sequencing Center, Baylor College of Medicine

^2^Dept. of Molecular and Human Genetics, Baylor College of Medicine

^3^Dept. of Evolutionary Anthropology, Duke University

^4^Dept. of Biological Sciences, Louisiana State University

^5^Dept. of Biology, Indiana University

^6^Dept. of Computer Science and Informatics, Indiana University

^7^Dept. of Biology, University of Bari, Bari, Italy

^8^Dept. of Genome Sciences, Univ. of Washington

^9^Dept. of Biochemistry and Molecular Genetics, Univ of Colorado Anschutz Medical Campus

^10^Institute of Population Genetics, University of Veterinary Medicine, Vienna

^11^ICREA at Institut de Biologia Evolutiva, Universitat Pompeu Fabra, Barcelona

^12^European Molecular Biology Laboratory, European Bioinformatics Institute, Hinxton

^13^Bioinformatics Research Center, Aarhus University, Aarhus

^14^Dept. of Biological Sciences, Norte Dame University

^15^Dept. of Anthropology, New York University

^16^Dept. of Genetics, Texas Biomedical Research Institute

^17^Emory University

^18^Cognitive Ethology Laboratory, German Primate Center, Gottingen

^19^Dept. of Biology, Univ. of Konstanz, Konstanz

^20^Primate Genetics Laboratory, German Primate Center, Gottingen

^21^Southwest National Primate Research Center, Texas Biomedical Research Institute

^22^Dept. of Physiology and Biophysics, Weill Cornell Medical College, New York and

HRH Prince Alwaleed Bin Talal Bin Abdulaziz Alsaud Inst. of Computational

Biomedicine, Weill Cornell Medicine

^23^Dept. of Neuroscience, Washington Univ. School of Medicine and Dept. of

Anthropology, Washington University

^24^Faculty of Mathematics, Physics and Informatics, Comenius University, Bratislava

^25^Dept. of Biology, University of Florence, Florence, Italy

^26^Howard Hughes Medical Institute
